# Epigenome-Wide Association Study Reveals Duration of Breastfeeding Is Associated with Epigenetic Differences in Children

**DOI:** 10.3390/ijerph17103569

**Published:** 2020-05-20

**Authors:** William B. Sherwood, Dilini M. Kothalawala, Latha Kadalayil, Susan Ewart, Hongmei Zhang, Wilfried Karmaus, S. Hasan Arshad, John W. Holloway, Faisal I. Rezwan

**Affiliations:** 1 Human Development and Health, Faculty of Medicine, University of Southampton, Southampton SO16 6YD, UK; ws2g14@soton.ac.uk (W.B.S.); d.kothalawala@soton.ac.uk (D.M.K.); lpk1r12@soton.ac.uk (L.K.); F.I.Rezwan@cranfield.ac.uk (F.I.R.); 2 NIHR Southampton Biomedical Research Centre, University Hospital Southampton NHS Foundation Trust, Southampton SO16 6YD, UK; S.H.Arshad@soton.ac.uk; 3 College of Veterinary Medicine, Michigan State University, East Lansing, MI 48824, USA; ewarts@msu.edu; 4 Division of Epidemiology, Biostatistics and Environmental Health, School of Public Health, University of Memphis, 236A Robison Hall, Memphis, TN 38152, USA; hzhang6@memphis.edu (H.Z.); karmaus1@memphis.edu (W.K.); 5 Clinical and Experimental Sciences, Faculty of Medicine, University of Southampton, Southampton SO16 6YD, UK; 6 The David Hide Asthma and Allergy Research Centre, St Mary’s Hospital, Isle of Wight PO30 5TG UK; 7 School of Water, Energy and Environment, Cranfield University, Cranfield, Bedfordshire MK43 0AL, UK

**Keywords:** Epigenome-Wide Association Study, EWAS, epigenetics, breastfeeding, breastfeeding duration, DNA methylation

## Abstract

Several small studies have shown associations between breastfeeding and genome-wide DNA methylation (DNAm). We performed a comprehensive Epigenome-Wide Association Study (EWAS) to identify associations between breastfeeding and DNAm patterns in childhood. We analysed DNAm data from the Isle of Wight Birth Cohort at birth, 10, 18 and 26 years. The feeding method was categorized as breastfeeding duration >3 months and >6 months, and exclusive breastfeeding duration >3 months. EWASs using robust linear regression were performed to identify differentially methylated positions (DMPs) in breastfed and non-breastfed children at age 10 (false discovery rate of 5%). Differentially methylated regions (DMRs) were identified using comb-p. The persistence of significant associations was evaluated in neonates and individuals at 18 and 26 years. Two DMPs, in genes *SNX25* and *LINC00840*, were significantly associated with breastfeeding duration >6 months at 10 years and was replicated for >3 months of exclusive breastfeeding. Additionally, a significant DMR spanning the gene *FDFT1* was identified in 10-year-old children who were exposed to a breastfeeding duration >3 months. None of these signals persisted to 18 or 26 years. This study lends further support for a suggestive role of DNAm in the known benefits of breastfeeding on a child’s future health.

## 1. Introduction

Early-life exposures are recognised as predictors of future health and development. The importance of diet in early childhood is well established. Breastfeeding is widely regarded as the best source of nutrition for infants [1], with the World Health Organisation (WHO) recommending exclusive breastfeeding for six months, after which weaning should occur [2]. Continued breastfeeding alongside complementary foods until two years of age is considered to offer further benefits.

The short- and long-term health advantages of breastfeeding have been clearly demonstrated, including a reduction in the risk of all-cause mortality [3], respiratory infections [4], childhood obesity [5], type-2 diabetes [6] and childhood leukaemia [7], though there are some inconsistencies between studies in the context of asthma [8]. Specific nutrients and maternal antibodies found in human breastmilk may explain some of these protective effects, particularly its short-term effects [9]. However, the mechanisms underlying the impact of breastfeeding on health throughout childhood, and extending into adulthood, remain largely unknown [10]. The role of breastfeeding in establishing the infant gut microbiome may be one important mechanism [11]. However, nutritional exposures in adults have shown to elicit epigenetic changes [12], generating the hypothesis that early life nutrition could cause long-term effects on health through epigenetic programming [13]. Long-lasting changes to human DNA methylation (DNAm) profiles have shown to be associated with early nutritional exposures [14,15,16]. As such, epigenetic processes have been proposed as a mechanism behind the long-term effects of breastfeeding [17].

A recent systematic review identified seven studies (five in humans and two in rats) where breastfeeding had been associated with DNAm, but their findings were inconclusive [18]. The studies were generally small and used different methods for epigenetic assessment, making comparison between the studies difficult. Rossnerova et al. measured DNAm in the peripheral blood of children (7–15 years) using the Illumina Infinium HumanMethylation27 BeadChip and concluded that the duration of exclusive breastfeeding was one factor that could impact DNAm patterns [19]. Obermann-Borst et al. used quantitative high-throughput mass spectrometry to measure DNAm at seven cytosine–phosphate–guanine (CpG) sites of the leptin gene (*LEP*), a gene proposed to be involved in the association between breastfeeding and childhood obesity [20]. The study showed that breastfeeding duration was significantly associated with a reduction of DNAm at those seven CpGs in *LEP* in 17-month-old infants. Further effects of breastfeeding duration have been observed in the infant metabolic epigenome [21] and with DNAm in immunoregulatory genes [22]. 

With the introduction of large-scale epigenetic profiling technologies, such as the Infinium HumanMethylation450 and Infinium MethylationEPIC (EPIC) BeadChips from Illumina, it has become possible to investigate the variation in DNAm on a genome-wide scale. A recent analysis of the relationship between breastfeeding duration and DNAm profiles of the *LEP* gene was conducted in 10-year-old children in the Isle of Wight Birth Cohort (IOWBC) [23]. The study identified an association between both total and exclusive breastfeeding duration (as a continuous variable) and DNAm at four CpG sites in the *LEP* locus in children at 10 years of age, but the association did not persist at 18 years. Additionally, in the same study, an Epigenome-Wide Association Study (EWAS) found breastfeeding duration to be associated with five differentially methylated regions (DMRs) at both 10 and 18 years [23]. However, no significant differentially methylated positions (CpGs) were observed to be associated with breastfeeding. With an increase in methylation data collected in the IOWBC, this study aims to address the limitations in the sample size and definition of breastfeeding exposure of the previous study. We performed a more comprehensive EWAS to identify the DNAm patterns in childhood associated with breastfeeding duration, and further analysed whether these signals persist into later life.

## 2. Materials and Methods 

### 2.1. Isle of Wight Birth Cohort

The Isle of Wight Birth Cohort (IOWBC), also known as the second generation, IoW F_1_, is a general population birth cohort (*n* = 1536) recruited between 1989 and 1990 [24,25]. The parents (first generation, IoW F_0_) of all infants born in the Isle of Wight over this period were contacted at birth and 1456 infants, for whom informed consent was obtained, were enrolled into the longitudinal study. Participants were followed up at 1 or 2, 4, 10, 18 and 26 years to collect information on allergic disease status and environmental exposures, including breastfeeding practice and infant nutrition [26]. Data on breastfeeding was available for 1332 participants. In addition, peripheral blood samples were collected at birth (neonatal heel prick on Guthrie cards) and at 10, 18 and 26 years. 

### 2.2. DNA Extraction and Microarray

DNA was extracted from peripheral blood samples using a standard salting-out procedure. Approximately 1 μg of DNA was bisulphite-treated following the EZ 96-DNA methylation kit (Zymo Research, Irvine, CA, USA) standard protocol. DNAm levels were measured for each sample using the Infinium MethylationEPIC BeadChips from Illumina (Illumina, San Diego, CA, USA) following the manufacturer’s standard protocol. DNAm data (β values) underwent pre-processing for quality control using the CPACOR package [27] and batch effect correction using ComBat [28]. Polymorphic and cross-hybridised probes were removed as described by McCartney et al. [29], leaving 538,693 CpGs for analysis.

DNAm data was available for 885, 410, 109 and 302 participants at birth, 10, 18 and 26 years, respectively. For singleton analysis, one participant was randomly removed from one pair of twins in the 10 years samples (*n* = 409). Participants with both phenotypic and DNAm data at 10 years (*n* = 356) were used for the EWAS, whilst such data at birth, 18 and 26 years were used for the follow-up analyses. 

### 2.3. Genotyping and Imputation

DNA from the blood samples (Isle of Wight Birth Cohort, *n* = 1101) were genotyped using the Illumina InfiniumOmni2.5-8v1.3 microarray. Standard quality control (QC) measures were applied to the genotype data to exclude samples with >3% missing genotypes, SNPs (single nucleotide polymorphisms) genotyped in <95% individuals, SNPs with minor allele frequencies (MAF) <0.5% and SNPs with significant deviations from Hardy–Weinberg equilibrium (*p*-value ≤ 1 × 10^−8^). Alleles in the quality-controlled genotype data were updated to match the direction (forward) and coordinates of those in the reference dataset GRCh37 [30]. A total of 1,641,983 SNPs and 1087 individuals were retained for imputation. Data were pre-phased (EAGLE2) [31] and imputed (PBWT) [32] at the Sanger Imputation Services (Oxford, UK). Data with an imputation quality >99% were retained. Further standard quality controls, including the removal of duplicate samples and SNPs, were performed to generate the final dataset containing 1071 samples and 5,031,289 SNPs for downstream analyses.

### 2.4. Categorisation of Breastfeeding Duration

For each participant, the exposure of breastfeeding duration was defined as the total number of weeks a mother breastfed her child, irrespective of the introduction of water, formula feeding and/or solid foods. Breastfeeding duration was categorised as >3 months and >6 months for the purpose of the primary analysis. For the 3 months category, participants were divided into two groups: never breastfed (*n* = 81) and breastfed >3 months (*n* = 163) (participants breastfed <3 months (*n* = 112) were excluded). Similarly, for the 6 months category, the sample sizes of the never breastfed and breastfed >6 months groups were 81 and 100, respectively.

For secondary analysis, a stringent exposure definition of exclusive breastfeeding duration was considered (*n* = 155). Exclusive breastfeeding duration was defined as the number of weeks a child was breastfed until the introduction of formula feed and/or solid foods. None of the participants were given water before this point. The exposed group consisted of those exclusively breastfed >3 months (*n* = 92). 

### 2.5. Confounding Factors

Environmental exposures known to influence both breastfeeding duration and DNAm were adjusted for in all association analyses performed in this study: sex, birthweight, maternal age, maternal smoking during pregnancy (reported at birth) and maternal socioeconomic status (defined using maternal socioeconomic cluster information based on household income, number of rooms and maternal education). Additionally, cell proportions (CD8T, CD4T, NK, B cells, monocytes and granulocytes) were estimated using the minfi package [33], with cell composition coefficients derived using the Houseman method [34].

### 2.6. Statistical Analyses

Two separate EWASs were performed to identify differentially methylated probes (DMPs) between exposed (>3 months and >6 months breastfeeding duration) and unexposed (never breastfed) subjects at age 10 using robust linear regression, adjusted for confounding factors and cell types. Multiple hypothesis testing was accounted for by controlling the false discovery rate (FDR) using Benjamini and Hochberg’s algorithm [35]. CpGs with an FDR-corrected *p*-value < 0.05 were considered statistically significant. 

Further, comb-p [36] was used to identify DMRs composed of multiple signals across individual CpG positions. Comb-p identifies regions enriched with low unadjusted *p*-values from the EWAS analysis. For each region, the DMR algorithm adjusts the CpG *p*-values for auto-correction between probes using the Stouffer–Liptak–Kechris (slk) correction, with multiple testing adjustments made using a one-step Sidak correction method. Regions with at least two CpG probes within 1000 base pairs, having a Sidak-corrected *p*-value < 0.05, were considered statistically significant.

DNAm levels of study participants who were never breastfed were compared with those breastfed >6 months across four different time points: shortly after birth (Guthrie cards (GU)) and at 10, 18 and 26 years of age. Only participants with both methylation and breastfeeding data were considered at each time point. In addition, each of the comparisons at birth, 18 and 26 years were performed on individuals that were also part of the comparison at the 10-year time point. Hence, the total number of participants analysed was Guthrie, *n* = 124; at 10 years, *n* = 181; at 18 years, *n* = 51; and at 26 years, *n* = 85 (Table S1). A nonparametric test, the Wilcoxon Rank Sum test, was used to compare the medians of the beta values for the exposed (>6 months of breastfeeding) and unexposed (no breastfeeding) groups at each of the four time points.

In a sensitivity analysis using the significant CpGs identified from the primary EWAS, non-breastfed controls (*n* = 63) were compared with participants who were exclusively breastfed >3 months (*n* = 92). A *cis*-mQTL analysis was conducted to test the association of the DNAm with nearby single nucleotide polymorphisms (SNPs), using the GEM package [37]. The region for the analysis was set within 50 kb upstream and downstream of each statistically significant CpG site. The model was adjusted using the same covariates as in the EWAS analysis, and significant associations were defined by an FDR-corrected *p*-value < 0.05. 

## 3. Results

Descriptive statistics comparing all participants in the IOWBC with DNAm data are presented in Table 1. The number of participants included in each step of the analysis is presented in Table S1.

### 3.1. EWAS of Breastfeeding Duration and DNA Methylation

**Figure 1:**
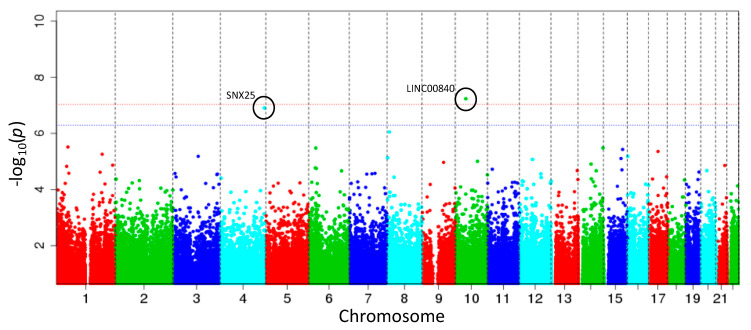
Genome-wide analysis of breastfeeding association with childhood DNAm. The Manhattan plot shows the association of breastfeeding duration >6 months and DNAm at 10 years. The model was adjusted for sex, birthweight, maternal age, maternal smoking at birth, maternal socioeconomic status and estimated cell type proportions. The blue and red lines indicate the FDR (false discovery rate) and Bonferroni (9.28 × 10^−8^) thresholds, respectively.

**Figure 2:**
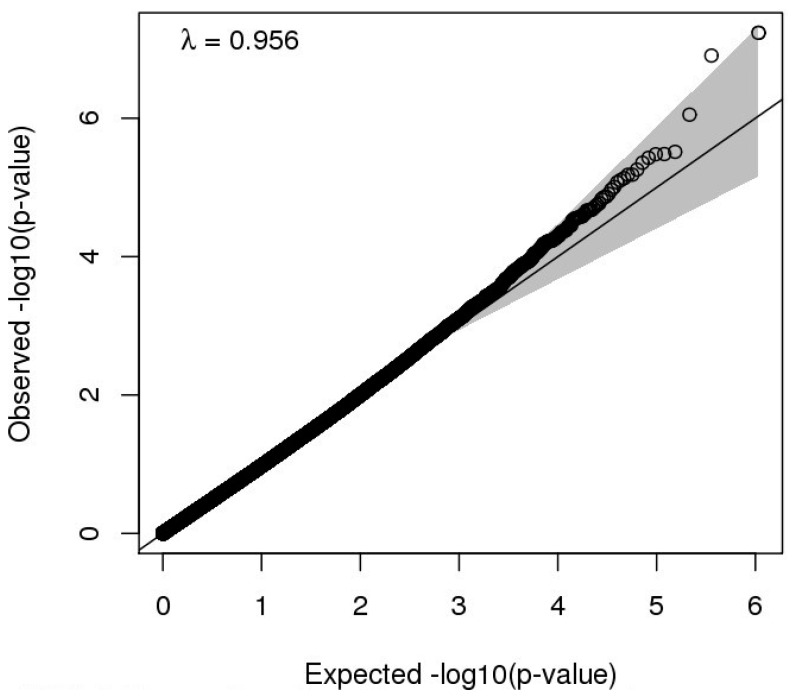
Quantile–Quantile (QQ) plot of the Epigenome-Wide Association Study (EWAS) for breastfeeding duration >6 months and DNAm in IOWBC at 10 years.

Breastfeeding duration >6 months was significantly associated with DNAm at two CpG sites at 10 years of age, with reduced methylation at cg03592955 and hypermethylation at cg08188863 (FDR < 0.05, Table 2 and Figure 1). A Quantile–Quantile (QQ) plot, used to indicate any deviations of the observed from the expected null distribution, showed no genomic inflation (λ = 0.956, Figure 2). Post-hoc power calculations for this EWAS indicate that 21.6% of the probes had >80% power to detect an effect of 2%. Mansell et al. highlight that a single power calculation may provide only limited information regarding the overall power of a DNAm study due to the variance of DNAm differing across probes in the array; they recommend calculating power at each individual site on the EPIC array [38]. The site-specific power to detect the effect sizes observed at each of the significant CpG sites (Table 2) was 3.58% and 0.54% for cg03592955 and cg08188863 (at a significance threshold of *p* = 9.42 × 10^−8^), respectively. The EWAS comparing DNAm at 10 years between participants never breastfed and breastfed >3 months did not identify any significant associations between breastfeeding duration >3 months and DNAm at any CpG sites (Supplementary Table S2 and Supplementary Figures S1 and S2).

### 3.2. Genome-Wide DMR Identifications

At age 10, the analysis of differentially methylated regions using comb-p for the breastfeeding duration >3 months category identified one significant DMR (Sidak-corrected *p*-value = 6.65 × 10^−5^) at chromosome eight (Table 3 and Supplementary Figure S3). No DMRs were identified for the >6 months breastfeeding duration category.

### 3.3. Persistence of DNA Methylation at Significant CpG Sites

**Figure 3:**
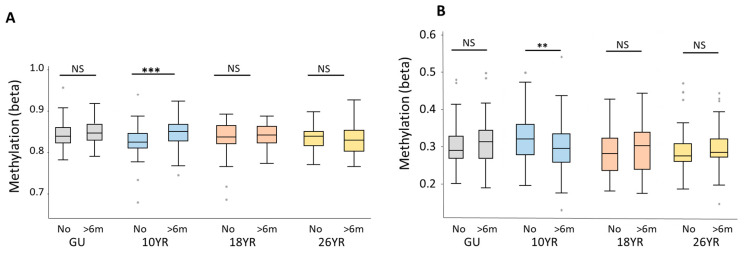
Influence of breastfeeding on the distribution of the beta values (methylation levels) at cg08188863 (*SNX25*) (**A**) and cg03592955 (*LINC00840*) (**B**) from birth to 26 years. Box plots show the distribution of the beta values measured from neonatal heel prick samples (GU) and blood samples collected at ages 10 (10YR), 18 (18YR) and 26 (26YR) years. At each of the four time points, the medians of the beta values for breastfed for greater than 6 months (>6m) and never breastfed (No) participants were compared. Wilcoxon Rank Sum *p*-values are reported. Sample sizes for the analysis for breastfed >6 months and never breastfed are, respectively, for GU: 65 vs. 59; 10YR: 101 vs. 83; 18YR: 29 vs. 22; and 26YR: 50 vs. 35. NS: not significant; *** indicates a *p*-value < 0.001 and ** indicates a *p*-value < 0.01.

The variation in DNAm levels (beta values) of cg08188863 and cg03592955 were compared in children across the four time points between birth and 26 years of age. For both CpGs, there was a statistically significant difference in the medians of the beta values between breastfed and non-breastfed children at 10 years of age (*p*-value = 0.006 and <0.0001 for cg08188863 and cg03592955, respectively; Figure 3, 10YR: No and >6 months). This is in line with the significant association of breastfeeding duration >6 months with cg08188863 and cg03592955 in 10-year-olds as identified from the EWAS. We hypothesized that the methylation levels for all neonates (Guthrie cards) would be similar, as these samples were collected soon after birth (within 2–3 days) and the infants were likely to have had minimal or no exposure to breastfeeding. As expected, no difference in the medians of the Guthrie beta values for either of the CpGs was observed (GU of Figure 3). However, no statistically significant differences in methylation levels were observed at either 18 or 26 years, suggesting that the associations identified at age 10 do not persist into adulthood (Figure 3, 18YR and 26YR).

### 3.4. EWAS of Exclusive Breastfeeding Duration and DNAm 

Both differentially methylated CpGs (cg08188863 and cg03592955) identified from the primary analysis in this study remained statistically significant with the more stringent exposure of exclusive breastfeeding over a shorter duration (>3 months, Table 4).

### 3.5. Association of Genotype and DNA Methylation

Cis-mQTL analysis was carried out in order to identify associations between the two significant CpG sites (cg03592955 and cg08188863) and SNPs located within 50 Kb upstream and downstream of the two CpGs (Supplementary Tables S3 and S4). No association was found between DNAm at the two CpGs and their nearby SNPs (FDR-corrected *p*-value > 0.05).

## 4. Discussion

In this study, we conducted an Epigenome-Wide Association Study to examine the association between breastfeeding duration and genome-wide DNAm profiles in 10-year-old children within the IOWBC. Two differentially methylated CpG sites were found to be significantly associated with breastfeeding duration >6 months (FDR-corrected *p*-value < 0.05). One of the CpG sites, cg08188863, found on the Sorting Nexin 25 (*SNX25*) gene, a protein involved in signalling and cargo sorting, was hypermethylated. Of its many functions, *SNX25* has been shown to downregulate transforming growth factor beta (TGF-β) signalling [39]. TGF-β is present in human breastmilk [40] and has been observed to be involved in the pathophysiology of a number of diseases, such as allergic and autoimmune diseases [41]. In addition, its role in epilepsy has also been described [42,43]. The risk of epilepsy in children has been associated with the duration of breastfeeding, with a longer duration of breastfeeding suggested to offer protective benefits [44]. The other significant CpG site, cg03592955, located on the long intergenic non-protein coding RNA 840 (*LINC00840*), was hypomethylated. However, post-hoc power calculations suggest that the site-specific power to detect the observed effect sizes in this analysis was only 4% and 1% for cg03592955 and cg08188863, respectively. This may be reflective of a truly small effect of breastfeeding on DNAm patterns 10 years later. It is possible that larger effects may be detected if DNAm was measured at earlier ages. However, due to the low power to detect significant differences in methylation of DMPs following correction for multiple testing, it is also possible that the two significant DMP associations detected were in fact false positive results. One differentially methylated region (DMR) was found to be significantly associated with the exposure of breastfeeding duration >3 months. This DMR harbours the gene, farnesyl-diphosphate farnesyltransferase 1 (*FDFT1*), which encodes a key enzyme, squalene synthase, involved in sterol biosynthesis. The expression of *FDFT1* has been found to be elevated in the visceral fat of individuals with abdominal obesity [45]. Inhibitors of *FDFT1* have been suggested for the treatment of hyperlipidaemia [46]. In addition, homozygous knock-out mice have shown that *FDFT1* plays an important role in cholesterol synthesis as well as the development of the central nervous system [47] and liver function [48]. Therefore, it is possible that breastfeeding may have positive influences on later life events, such as obesity, heart disease and neurological function, via methylation at these two CpG sites.

The differential methylation at the two CpG sites observed at 10 years of age did not persist in a subset of individuals with DNAm data at 18 and 26 years. Whilst the association observed at 10 years may be suggestive of only a short-term effect of breastfeeding on DNAm across an individual’s lifespan, this analysis may have been limited by the small sample size at each of the later time points. 

By using an exposure definition of breastfeeding duration, it is possible that the results were confounded by other early life feeding modalities. To address this, we used a more stringent definition of exclusive breastfeeding. The significant associations found were replicated for this exposure at a shorter duration of exclusive breastfeeding for >3 months, suggesting the associations observed for >6 months breastfeeding duration were due to breastfeeding and not confounded by the introduction of supplemental feeding or solid food. The lack of difference of methylation at these CpGs in the DNAm profiles of individuals at birth (Guthrie cards) also provides support for a potential true effect of breastfeeding on DNAm.

Whilst our study detected an association between DNAm and breastfeeding, it is difficult to determine causality. Given that other factors could influence changes in DNAm, it is difficult to quantify the true contribution of breastfeeding on DNAm. To address this, we adjusted for all known confounders in our EWAS analyses. We further conducted cis-mQTL analyses, which revealed that changes in DNAm were not affected by genetic variations (SNPs). Therefore, potential mechanisms of breastfeeding driving changes in DNAm may include either the direct effects of nutritional and bioactive factors found in breastmilk, or indirect maternal nurturing behaviours, or both [49]. 

As our collective understanding of the role of epigenetics in human disease grows, so does the implications for public health. It has become apparent that, based on how we live our lives, environmental exposures and disease may have lasting repercussions for the health of our children and even grandchildren through epigenetic programming. Moreover, early life exposures seem to have a strong effect on DNA methylation. Consequently, understanding the potential mechanistic role of DNAm in the long-term effects of breastfeeding is valuable from a public health perspective in the promotion of breastfeeding and associated risk assessment [50]. Our findings suggest that breastfeeding may exert long-term effects on health and, although these changes in DNAm appear small, they may be functionally significant. Future research is required to establish (i) the degree of DNAm that can be considered clinically significant; (ii) the role of DNAm across cohorts with different demographic characteristics; and (iii) the variability in DNAm throughout childhood. Furthermore, a more conclusive “dose–response” assessment is important to support the ongoing development of breastfeeding/infant nutrition guidelines. Finally, identifying the key components of breastmilk that may be responsible for these long-term beneficial effects may promote improved engineering of formula feeds in the future.

There were limitations to this study. Primarily, power was a significant issue. Whilst two CpG sites were significantly associated with breastfeeding >6 months, the low site-specific power reported limits our confidence that these findings are true and can be replicated. Similarly, the substantially lower sample size at the 18-year time point compared to at 10 years is likely to have prevented any significant results for the persistence of DNAm, if present, to be detected. Therefore, it is clear that external replication is required to validate these results. Ideally, future studies should meta-analyse data from multiple cohorts in order to avoid their studies being hindered by low power and sample size. Indeed, recently, two Epigenome-Wide Association Studies have been conducted in children from buccal cells collected around age nine [51] and peripheral blood cells at ages 7 and 15–17 [52]. Both studies reported some evidence for the association of breastfeeding with offspring DNA methylation. Although none of the identified DMPs or DMRs overlap with those identified in this study, the differences in tissue, array platform, exposure definition and age of assessment make direct comparisons difficult.

## 5. Conclusions

This study has provided further evidence to support a role for breastfeeding in shaping a child’s epigenome. We identified two CpG sites that were significantly associated with breastfeeding duration >6 months in 10-year-old children. One of these sites, located on *SNX25*, may be involved in the regulation of TGF-β signalling and, subsequently, the potential development of later life diseases. However, these associations did not persist into adulthood. In addition, a single DMR spanning *FDFT1*, a gene in the cholesterol biosynthesis pathway, was also associated with breastfeeding duration >3 months. These associations warrant further investigation. As already implemented for a number of EWAS analyses of childhood epigenetics [53], a single and sufficiently powered multi-cohort analysis may offer the best opportunity to detect associations between breastfeeding and DNA methylation with high confidence. 

## Figures and Tables

**Table 1:** Baseline characteristics of participants in the Isle of Wight Birth Cohort (IOWBC) with DNAm data at each time point.

Characteristics	IOWBC (*n* = 1536)	Guthrie (*n* = 885)	10 Years (*n* = 410)	18 Years (*n* = 109)	26 Years (*n* = 302)
Child sex (female)	750 (48.8%)	457 (51.6%)	169 (41.2%)	4 (3.7%)	171 (56.6%)
Low birth weight (<2500 g)	64 (4.2%)	32 (3.6%)	16 (3.9%)	6 (5.5%)	15 (5.0%)
Socioeconomic status					
1 (lowest)	209 (13.6%)	121 (13.7%)	52 (12.7%)	11 (10.1%)	46 (15.2%)
2	240 (15.6%)	154 (17.4%)	78 (19.0%)	20 (18.3%)	60 (19.9%)
3	403 (26.2%)	299 (33.8%)	126 (30.7%)	36 (33.0%)	84 (27.8%)
4	394 (25.7%)	220 (24.9%)	114 (27.8%)	34 (31.2%)	82 (27.2%)
5 (highest)	111 (7.2%)	70 (7.9%)	37 (9.0%)	7 (6.4%)	23 (7.6%)
Maternal Smoking	384 (25.0%)	185 (20.9%)	80 (19.5%)	19 (17.4%)	58 (19.2%)
Maternal age (mean, standard deviation)	26.77, 5.36	26.78, 5.16	27.06, 5.19	26.89, 4.84	26.86, 5.11
Never breastfed	358 (23.3%)	599 (67.7%)	83 (20.2%)	23 (21.1%)	49 (16.2%)

**Table 2:** Association between the DNAm of the top five CpG sites with breastfeeding duration >6 months at 10 years.

CpG	Chr	Map Info	UCSC Gene Name	Beta	SE	*p*-Value *	FDR-Adjusted *p*-Value ^†^
cg03592955	10	44373919	*LINC00840*	−0.032	0.006	5.82 × 10^−8^	**0.019**
cg08188863	4	186253778	*SNX25*	0.024	0.005	1.24 × 10^−7^	**0.020**
cg25268605	1	47698518	*TAL1*	−0.037	0.008	3.05 × 10^−6^	0.241
cg04957663	6	29587487	*GABBR1*	−0.016	0.004	3.32 × 10^−6^	0.241
cg14723566	15	80711027	*ARNT2*	−0.011	0.002	3.73 × 10^−6^	0.241

Only the top five CpG sites are included, see Supplementary Table S3, for the top 50 sites. * *p*-values given are raw (FDR-unadjusted). ^†^ FDR-adjusted *p*-values < 0.05 were considered significant (in bold). CpG: name of the CpG site; Chr: chromosome; Map Info: genomic location of each site in genome build GRCh37; Beta: regression coefficient; SE: standard error of the regression coefficient; FDR: False Discovery Rate.

**Table 3:** Statistically significant differentially methylated regions (DMRs) (Sidak *p*-value < 0.05) for breastfeeding duration >3 months.

Location	No. of Probes	Slk *p*-Value	Sidak *p*-Value	Ref. Gene Name and Genomic Feature		CpG Feature
chr8: 11666256–11666619	6	9.44 × 10^−10^	6.65 × 10^−5^	*FDFT1*	S_Shore	NA

*Slk p*-value: uncorrected Stouffer–Liptak–Kechris *p*-values; Sidak *p*-value: corrected for multiple testing; S_Shore: South shore of CpG Island; NA: not available.

**Table 4:** Exclusive breastfeeding duration >3 months vs. DNAm at cg03592955 and cg08188863 at 10 years.

CpG	Chr	Map Info	UCSC Gene Name	Beta	SE	*p*-Value *
cg03592955	10	44373919	*LINC00840*	−0.019	0.006	0.002
cg08188863	4	186253778	*SNX25*	0.010	0.005	0.028

* *p*-values < 0.05 are considered significant (shown in bold). CpG: name of the CpG site; Chr: chromosome; Map Info: genomic location of each site in genome build GRCh37; Beta: regression coefficient; SE: standard error of the regression coefficient.

## Supplementary Materials

The following are available online at https://www.mdpi.com/1660-4601/17/10/3569/s1, Table S1. Summary of the number of participants included in each analysis in this study; Table S2. Breastfeeding duration >3 months vs. DNA methylation at 10 years (top 10 hits); Table S3. Cis-mQTL analysis results for cg03592955; Table S4. Cis-mQTL analysis results for cg08188863; Figure S1: Manhattan plot of the EWAS on breastfeeding duration >3 months and DNAm in the IOWBC at 10 years; Figure S2: Quantile–Quantile (QQ) plot of the EWAS on breastfeeding duration >3 months and DNAm in the IOWBC at 10 years; Figure S3. Manhattan plot for the DMR analysis.
