# Supplementary material for: Epigenome-Wide Association Study Reveals Duration of Breastfeeding Is Associated with Epigenetic Differences in Children
Source: Int J Environ Res Public Health. 2020 May 20;17(10):3569. doi: 10.3390/ijerph17103569 (PMC7277240; doi:10.3390/ijerph17103569)
Supplement: Supplementary file 1 [file ijerph-17-03569-s001.pdf]

Supplementary materials

Table 1. Summary of the number of participants included in each analysis in this study.

	Analysis	Guthrie n (% exposed)	10YR *n* (% exposed)	18YR *n* (% exposed)	26YR *n* (% exposed)
Primary analyses	EWAS: Exposure: No breastfeeding vs breastfeeding duration >3 months	-	244 (66.80%)	-	-
	EWAS: Exposure: No breastfeeding vs breastfeeding duration >6 months	-	181(55.25%)	-	-
Secondary analyses	EWAS: Exposure: No breastfeeding vs exclusive breastfeeding duration >3 months	-	155 (59.35%)	-	-
	Persistence of DNA methylation at significant CpG sites	124 (52.42)	184 (55.80%)	51 (52.72%)	85 (62.50%)
	mQTL analysis: For significant sites identified from the primary EWASs	-	181(55.25%)	-	-

Table 2. Breastfeeding duration >3 months vs DNA methylation at 10 years (top 10 hits).

Illumina ID	Chr	Map info	UCSC Gene name	Beta	SE	*p*-value	Adj *p*-value
cg04168168	11	115860944		-0.03495273	0.007243702	1.40E-06	0.460237766
cg22992837	11	113643339	ZW10	0.02113391	0.004637724	5.19E-06	0.568278858
cg14361688	10	70516151	CCAR1	0.01378314	0.003061900	6.75E-06	0.568278858
cg11021551	7	108097740	NRCAM	0.02634006	0.005857811	6.91E-06	0.568278858
cg16101148	12	59990794		0.005231706	0.001208083	1.49E-05	0.640836667
cg08188863	4	186253778	SNX25	0.01848824	0.004276839	1.54E-05	0.640836667
cg24717556	1	179834809	TOR1AIP2	-0.00974469	0.002283334	1.97E-05	0.640836667
cg16150003	12	54556746		-0.01260559	0.002955751	2.00E-05	0.640836667
cg01284406	2	209271151	PTH2R	-0.02675215	0.006323477	2.33E-05	0.640836667
cg22218827	8	1712650	CLN8	-0.00543326	0.001284788	2.35E-05	0.640836667

Columns: Illumina ID = EPIC CpG site numbers; Chr = chromosomal location of each site; Map info = genomic location of each site; UCSC gene name; beta = regression coefficient; SE = standard error of the regression coefficient; raw p-value; Adj p-value = p-value<0.05 following FDR correction was considered significant. Top 50 hits.

Table 3. Cis-mQTL analysis results for cg03592955.

chr	Position	SNP	ref	alt	beta	*p*-value	FDR	Distance
10	44380000	rs13313099	C	T	0.017	0.011	0.849	6081
10	44368244	rs10899926	T	C	0.016	0.013	0.849	-5675
10	44373172	rs12242985	A	G	0.016	0.013	0.849	-747
10	44373858	rs10899930	A	G	0.016	0.014	0.849	-61
10	44374085	rs10899931	A	C	0.016	0.014	0.849	166
10	44382946	rs2224985	A	G	0.016	0.014	0.849	9027
10	44383053	rs10899938	C	A	0.016	0.014	0.849	9134
10	44385605	rs7097229	G	T	0.016	0.014	0.849	11686
10	44386102	rs1947672	G	A	0.016	0.014	0.849	12183
10	44375925	rs10899935	T	G	0.016	0.018	0.849	2006
10	44359262	rs11238747	C	T	0.016	0.023	0.849	-14657
10	44371316	rs141818853	C	T	0.016	0.023	0.849	-2603
10	44375912	rs11238765	T	G	0.016	0.023	0.849	1993
10	44376051	rs10899936	G	A	0.016	0.023	0.849	2132
10	44378740	rs7097453	G	A	0.016	0.023	0.849	4821
10	44379136	rs1325490	T	C	0.016	0.023	0.849	5217
10	44380589	rs7086120	T	C	0.016	0.023	0.849	6670
10	44381350	rs73277915	G	C	0.016	0.023	0.849	7431
10	44381492	rs12248836	A	G	0.016	0.023	0.849	7573
10	44384897	rs1460540	A	C	0.016	0.023	0.849	10978
10	44386566	rs10899939	T	C	0.016	0.023	0.849	12647
10	44388083	rs10899940	G	C	0.016	0.023	0.849	14164
10	44390503	rs10899941	C	G	0.016	0.023	0.849	16584
10	44390863	rs10899942	A	G	0.016	0.023	0.849	16944
10	44355590	rs11238745	G	C	0.015	0.026	0.849	-18329
10	44375635	rs10899933	T	C	0.014	0.029	0.849	1716
10	44375680	rs10899934	G	C	0.014	0.029	0.849	1761
10	44376927	rs7073044	C	T	0.014	0.029	0.849	3008
10	44375264	rs7901541	T	C	0.014	0.035	0.849	1345
10	44352374	rs118162120	C	T	0.013	0.048	0.849	-21545
10	44401456	rs11238771	G	C	0.015	0.053	0.849	27537
10	44405997	rs7914991	T	C	0.015	0.053	0.849	32078
10	44406205	rs10899949	G	A	0.015	0.053	0.849	32286
10	44420538	rs12241154	C	T	0.015	0.053	0.849	46619
10	44423144	rs11238779	A	T	0.015	0.053	0.849	49225
10	44400396	rs10793506	G	C	0.006	0.062	0.849	26477
10	44345353	rs74420036	T	C	0.012	0.067	0.849	-28566
10	44345430	rs80145431	G	C	0.012	0.067	0.849	-28489
10	44399052	rs10899947	C	T	0.006	0.072	0.849	25133
10	44422032	rs10793507	G	C	0.006	0.072	0.849	48113
10	44336374	rs7901526	C	A	-0.006	0.077	0.849	-37545
10	44343087	rs748715	T	C	-0.006	0.090	0.849	-30832
10	44418600	rs2147876	T	C	0.006	0.106	0.849	44681
10	44418619	rs2147875	C	T	0.006	0.106	0.849	44700
10	44418891	rs1147902	G	A	0.006	0.106	0.849	44972
10	44421479	rs1254744	G	A	0.006	0.106	0.849	47560
10	44422688	rs1254855	T	C	0.006	0.106	0.849	48769
10	44423334	rs1254857	T	G	0.006	0.106	0.849	49415
10	44371307	rs138765708	T	A	0.019	0.112	0.849	-2612
10	44396631	rs898548	A	G	0.006	0.114	0.849	22712
10	44400326	rs1303233	G	C	0.006	0.114	0.849	26407
10	44402377	rs1570847	T	C	0.006	0.114	0.849	28458
10	44402926	rs2169198	T	C	0.006	0.114	0.849	29007
10	44394825	rs7920691	T	C	0.012	0.128	0.849	20906

10	44347970	rs11238741	T	C	-0.005	0.130	0.849	-25949
10	44373192	rs10899929	A	G	-0.005	0.132	0.849	-727
10	44347246	rs4948580	T	A	-0.005	0.140	0.849	-26673
10	44338535	rs4948784	A	G	-0.005	0.142	0.849	-35384
10	44365019	rs2031513	G	T	-0.005	0.147	0.849	-8900
10	44366841	rs11238753	A	G	-0.005	0.147	0.849	-7078
10	44367155	rs11238754	T	C	-0.005	0.147	0.849	-6764
10	44369899	rs10899927	T	C	-0.005	0.147	0.849	-4020
10	44370755	rs11238759	C	T	-0.005	0.147	0.849	-3164
10	44370872	rs11238761	C	T	-0.005	0.147	0.849	-3047
10	44347063	rs7909314	A	G	-0.005	0.149	0.849	-26856
10	44348098	rs1468061	A	G	-0.005	0.149	0.849	-25821
10	44349516	10:44349516:GC	G	C	-0.005	0.149	0.849	-24403
10	44358219	rs2169200	A	C	-0.005	0.150	0.849	-15700
10	44361284	rs1009770	T	C	-0.005	0.150	0.849	-12635
10	44362231	rs11238749	T	C	-0.005	0.150	0.849	-11688
10	44362266	rs11238750	A	G	-0.005	0.150	0.849	-11653
10	44364706	rs912805	C	T	-0.005	0.155	0.849	-9213
10	44365238	rs4948581	G	C	-0.005	0.155	0.849	-8681
10	44365540	rs10899925	C	T	-0.005	0.155	0.849	-8379
10	44366565	rs12359360	C	T	-0.005	0.155	0.849	-7354
10	44367287	rs11238756	C	T	-0.005	0.155	0.849	-6632
10	44369906	rs10899928	G	A	-0.005	0.155	0.849	-4013
10	44358300	rs1008803	G	A	-0.005	0.158	0.849	-15619
10	44360599	rs9633744	C	A	-0.005	0.158	0.849	-13320
10	44361106	rs1009771	G	A	-0.005	0.158	0.849	-12813
10	44361352	rs1009769	C	T	-0.005	0.158	0.849	-12567
10	44371903	rs7903927	G	A	-0.005	0.160	0.849	-2016
10	44384191	rs11238766	C	T	-0.005	0.160	0.849	10272
10	44390053	rs56034709	G	A	-0.005	0.165	0.849	16134
10	44340493	rs11238739	T	C	-0.005	0.166	0.849	-33426
10	44340865	rs4948785	A	G	-0.005	0.166	0.849	-33054
10	44344738	rs10793503	C	T	-0.005	0.167	0.849	-29181
10	44341964	rs10899922	G	A	-0.005	0.168	0.849	-31955
10	44353871	rs881714	A	C	-0.005	0.176	0.849	-20048
10	44330344	rs2863232	A	G	0.005	0.176	0.849	-43575
10	44339418	rs11238738	G	A	-0.005	0.180	0.849	-34501
10	44327234	rs189730164	C	T	-0.011	0.182	0.849	-46685
10	44327423	rs138650915	A	G	-0.011	0.182	0.849	-46496
10	44333255	rs77211970	G	T	-0.011	0.182	0.849	-40664
10	44335562	rs79746840	A	G	-0.011	0.182	0.849	-38357
10	44338565	rs112414482	T	C	-0.011	0.182	0.849	-35354
10	44339185	rs41531444	A	G	-0.011	0.182	0.849	-34734
10	44340062	rs78964431	T	C	-0.011	0.182	0.849	-33857
10	44342146	rs73275930	A	T	-0.011	0.182	0.849	-31773
10	44352569	rs4948786	A	G	-0.005	0.185	0.849	-21350
10	44357854	rs10899923	C	T	-0.005	0.185	0.849	-16065
10	44337215	rs1870636	T	G	-0.005	0.198	0.849	-36704
10	44418721	rs2613103	A	C	-0.010	0.214	0.849	44802
10	44418020	rs10899950	T	C	-0.004	0.220	0.849	44101
10	44337098	rs11238736	T	G	-0.004	0.225	0.849	-36821
10	44337481	rs10899921	T	C	-0.004	0.225	0.849	-36438
10	44338110	rs11238737	A	G	-0.004	0.225	0.849	-35809
10	44345134	rs78197031	G	A	-0.011	0.241	0.849	-28785
10	44395825	rs883400	G	T	0.004	0.242	0.849	21906
10	44405852	rs75554270	A	G	-0.007	0.242	0.849	31933
10	44335624	rs80307761	T	C	-0.008	0.245	0.849	-38295

10	44415929	rs11238774	T	G	-0.004	0.247	0.849	42010
10	44416074	rs12359315	T	C	-0.004	0.247	0.849	42155
10	44400595	rs10751347	T	C	0.004	0.248	0.849	26676
10	44392564	rs77100892	C	A	0.009	0.250	0.849	18645
10	44409934	rs76647584	G	A	-0.006	0.265	0.849	36015
10	44414610	rs4948790	T	C	-0.006	0.265	0.849	40691
10	44347103	rs77452812	A	G	-0.009	0.271	0.849	-26816
10	44350037	rs17154506	G	A	-0.009	0.271	0.849	-23882
10	44354068	rs78336318	T	G	-0.009	0.271	0.849	-19851
10	44360748	rs78359121	C	T	-0.009	0.271	0.849	-13171
10	44364875	10:44364875:AG	A	G	-0.009	0.271	0.849	-9044
10	44366402	rs59230148	A	G	-0.009	0.271	0.849	-7517
10	44396830	rs2290877	A	T	-0.006	0.272	0.849	22911
10	44404397	rs55901602	A	C	-0.006	0.272	0.849	30478
10	44398862	rs1468060	A	G	-0.012	0.273	0.849	24943
10	44399660	rs1147901	G	C	-0.009	0.274	0.849	25741
10	44403820	rs1749801	G	C	-0.009	0.274	0.849	29901
10	44404001	rs1778431	T	G	-0.009	0.274	0.849	30082
10	44408026	rs2798991	A	C	-0.009	0.274	0.849	34107
10	44408190	rs2798992	A	G	-0.009	0.274	0.849	34271
10	44411868	rs1254852	T	C	-0.009	0.274	0.849	37949
10	44416237	rs1254850	T	G	-0.009	0.274	0.849	42318
10	44422512	rs1270513	C	T	-0.009	0.274	0.849	48593
10	44423030	rs1254856	A	G	-0.009	0.274	0.849	49111
10	44399192	rs7900414	A	C	0.004	0.294	0.849	25273
10	44400997	rs10508874	C	T	0.004	0.294	0.849	27078
10	44401195	rs10899948	A	G	0.004	0.294	0.849	27276
10	44403522	rs10751348	G	A	0.004	0.294	0.849	29603
10	44403834	rs4948787	A	G	0.004	0.294	0.849	29915
10	44405258	rs882902	T	C	0.004	0.294	0.849	31339
10	44406302	rs7090750	G	T	0.004	0.294	0.849	32383
10	44407430	rs1813245	A	G	0.004	0.294	0.849	33511
10	44407897	rs2274241	T	G	0.004	0.294	0.849	33978
10	44410434	rs9732790	T	C	0.004	0.294	0.849	36515
10	44416505	rs1813897	C	T	0.004	0.294	0.849	42586
10	44338657	rs7918642	G	A	-0.007	0.295	0.849	-35262
10	44398291	10:44398291:CT	T	C	0.004	0.297	0.849	24372
10	44418976	rs10899951	A	T	-0.003	0.302	0.849	45057
10	44422554	rs11238778	A	G	-0.003	0.302	0.849	48635
10	44324537	rs4948780	T	C	0.003	0.318	0.849	-49382
10	44324621	rs7094300	G	A	0.003	0.318	0.849	-49298
10	44325795	rs11238725	A	G	0.003	0.318	0.849	-48124
10	44327128	rs10899919	G	A	0.003	0.324	0.849	-46791
10	44327192	rs78973389	A	G	0.003	0.324	0.849	-46727
10	44327434	rs11238728	G	A	0.003	0.324	0.849	-46485
10	44327625	rs11238729	T	C	0.003	0.324	0.849	-46294
10	44398861	rs7915088	C	T	0.003	0.335	0.849	24942
10	44394568	rs1147900	C	G	0.003	0.367	0.849	20649
10	44375162	rs11238764	T	C	-0.003	0.372	0.849	1243
10	44401706	rs117615891	A	G	-0.009	0.415	0.849	27787
10	44329329	rs11238731	T	C	0.003	0.420	0.849	-44590
10	44375711	rs17465611	A	C	-0.003	0.426	0.849	1792
10	44376756	rs10899937	T	C	-0.003	0.426	0.849	2837
10	44330276	rs11238733	C	G	0.003	0.426	0.849	-43643
10	44330428	rs2147873	T	C	0.003	0.426	0.849	-43491
10	44330934	rs7913475	T	C	0.003	0.426	0.849	-42985
10	44331679	rs4350323	T	G	0.003	0.426	0.849	-42240

10	44331803	rs4429020	A	G	0.003	0.426	0.849	-42116
10	44332080	rs2085798	C	T	0.003	0.426	0.849	-41839
10	44394465	rs1617058	A	G	0.003	0.431	0.849	20546
10	44394466	rs1617059	C	T	0.003	0.431	0.849	20547
10	44363286	rs997193	T	C	0.003	0.439	0.855	-10633
10	44339315	rs1675291	T	C	0.004	0.441	0.857	-34604
10	44347717	rs10793504	T	A	0.003	0.456	0.863	-26202
10	44354542	rs61859388	C	A	0.005	0.469	0.863	-19377
10	44350464	rs1078113	C	G	0.002	0.513	0.890	-23455
10	44422595	rs75463954	A	T	-0.013	0.526	0.890	48676
10	44365715	rs1547163	A	G	0.002	0.540	0.896	-8204
10	44370167	rs7070116	A	G	0.002	0.540	0.896	-3752
10	44358550	rs1008804	G	A	0.002	0.550	0.903	-15369
10	44359632	rs10899924	T	C	0.002	0.550	0.903	-14287
10	44361608	rs11238748	A	G	0.002	0.550	0.903	-12311
10	44362711	rs7921870	T	C	0.002	0.550	0.903	-11208
10	44391001	rs1578854	T	G	0.002	0.569	0.910	17082
10	44374899	rs7920511	T	C	-0.002	0.612	0.910	980
10	44375543	rs10751346	C	T	-0.002	0.612	0.910	1624
10	44374786	rs74138916	A	G	-0.009	0.696	0.945	867
10	44377358	rs117813471	A	T	-0.009	0.696	0.945	3439
10	44389741	rs142552003	T	C	-0.009	0.696	0.945	15822
10	44391094	rs77192027	A	G	-0.009	0.696	0.945	17175
10	44391497	rs74878412	C	T	-0.009	0.696	0.945	17578
10	44391565	rs75464377	C	T	-0.009	0.696	0.945	17646
10	44391945	rs117898551	A	C	-0.009	0.696	0.945	18026
10	44358747	rs75419445	A	G	-0.005	0.717	0.945	-15172
10	44376205	rs7921967	A	G	-0.001	0.723	0.945	2286
10	44403927	rs75541195	T	C	-0.006	0.733	0.945	30008
10	44375239	rs2613102	T	C	-0.001	0.818	0.945	1320
10	44328564	rs142522109	T	C	0.001	0.826	0.945	-45355
10	44384758	rs12218783	C	T	-0.004	0.830	0.945	10839
10	44346094	rs1254846	G	A	0.001	0.856	0.945	-27825
10	44392411	rs7893153	A	G	0.001	0.861	0.947	18492
10	44365497	rs79288982	T	C	-0.004	0.862	0.947	-8422
10	44383342	rs117503595	A	G	-0.004	0.862	0.947	9423
10	44392068	rs7895918	T	C	0.000	0.887	0.950	18149
10	44337768	rs117030511	T	C	-0.003	0.895	0.950	-36151
10	44337849	rs76720082	A	G	-0.003	0.895	0.950	-36070
10	44339911	rs58426522	G	C	-0.003	0.895	0.950	-34008
10	44340199	rs74862729	G	A	-0.003	0.895	0.950	-33720
10	44343871	rs78864401	A	G	-0.003	0.895	0.950	-30048
10	44344612	rs74873320	A	G	-0.003	0.895	0.950	-29307
10	44345432	rs75148678	T	C	-0.003	0.895	0.950	-28487
10	44346388	rs117769578	G	C	-0.003	0.895	0.950	-27531
10	44348379	rs59274145	A	G	-0.003	0.895	0.950	-25540
10	44349049	rs78226429	T	C	-0.003	0.895	0.950	-24870
10	44349238	rs74828131	A	G	-0.003	0.895	0.950	-24681
10	44349483	rs80183712	T	C	-0.003	0.895	0.950	-24436
10	44349580	rs78129090	A	G	-0.003	0.895	0.950	-24339
10	44350143	rs78158539	G	T	-0.003	0.895	0.950	-23776
10	44351049	rs57882812	G	C	-0.003	0.895	0.950	-22870
10	44351411	rs116978589	A	T	-0.003	0.895	0.950	-22508
10	44352094	rs78554189	C	T	-0.003	0.895	0.950	-21825
10	44352463	rs115566767	A	G	-0.003	0.895	0.950	-21456
10	44355400	rs713496	T	C	-0.003	0.895	0.950	-18519
10	44356010	rs79958406	T	G	-0.003	0.895	0.950	-17909

10	44356323	rs76846594	C	T	-0.003	0.895	0.950	-17596
10	44356828	rs58838440	A	G	-0.003	0.895	0.950	-17091
10	44357126	rs59983288	G	T	-0.003	0.895	0.950	-16793
10	44357192	rs58526299	G	C	-0.003	0.895	0.950	-16727
10	44357249	rs59668941	G	A	-0.003	0.895	0.950	-16670
10	44358117	rs61525746	A	C	-0.003	0.895	0.950	-15802
10	44360132	rs77229889	G	T	-0.003	0.895	0.950	-13787
10	44360297	rs79466182	T	C	-0.003	0.895	0.950	-13622
10	44361562	rs75728939	T	C	-0.003	0.895	0.950	-12357
10	44362681	rs60833858	G	T	-0.003	0.895	0.950	-11238
10	44362721	rs60927183	T	C	-0.003	0.895	0.950	-11198
10	44364477	rs79823602	A	G	-0.003	0.895	0.950	-9442
10	44364804	rs57645083	C	T	-0.003	0.895	0.950	-9115
10	44365368	rs76777017	T	C	-0.003	0.895	0.950	-8551
10	44365656	rs79332046	C	T	-0.003	0.895	0.950	-8263
10	44365762	rs73275973	A	C	-0.003	0.895	0.950	-8157
10	44367818	rs75486433	T	C	-0.003	0.895	0.950	-6101
10	44368138	rs73275981	C	T	-0.003	0.895	0.950	-5781
10	44369284	rs56942692	A	G	-0.003	0.895	0.950	-4635
10	44370483	rs73275989	C	A	-0.003	0.895	0.950	-3436
10	44370723	rs73275990	G	T	-0.003	0.895	0.950	-3196
10	44371229	rs118076558	T	C	-0.003	0.895	0.950	-2690
10	44371674	rs2298204	G	A	-0.003	0.895	0.950	-2245
10	44372082	rs78275993	T	C	-0.003	0.895	0.950	-1837
10	44372085	rs75944252	T	A	-0.003	0.895	0.950	-1834
10	44372318	rs73275995	A	G	-0.003	0.895	0.950	-1601
10	44373377	rs73275997	C	T	-0.003	0.895	0.950	-542
10	44373540	rs73275998	T	C	-0.003	0.895	0.950	-379
10	44373721	rs78155419	T	C	-0.003	0.895	0.950	-198
10	44334916	rs11238734	T	G	0.001	0.903	0.950	-39003
10	44325941	rs61859379	G	A	0.000	0.923	0.958	-47978
10	44329569	rs117046105	T	C	0.000	0.923	0.958	-44350
10	44329743	rs61859383	T	C	0.000	0.923	0.958	-44176
10	44332603	rs1841390	G	A	0.000	0.923	0.958	-41316
10	44388243	rs78242607	T	G	0.000	0.939	0.961	14324
10	44391132	rs7096312	G	A	0.000	0.939	0.961	17213
10	44367267	rs11238755	C	A	0.000	0.952	0.970	-6652
10	44375320	10:44375320:TC	T	C	0.001	0.980	0.988	1401

chr = chromosome, position = position of the SNP, SNP = SNP ID, ref = reference allele, alt = alternate allele, beta = regression coefficient, FDR = false discovery rate adjusted p-value, distance = distance of the SNP from the CpG site.

Table 4. Cis-mQTL analysis results for cg08188863.

chr	Position	SNP	ref	alt	beta	*p*-value	FDR	Distance
4	186203808	rs67747917	T	C	0.0072	0.062	0.728	-49970
4	186216899	rs115315139	T	C	-0.0217	0.120	0.728	-36879
4	186299376	rs75948372	G	A	-0.0118	0.178	0.762	45598
4	186300451	rs78919784	A	G	-0.0118	0.178	0.762	46673
4	186303517	rs75930868	G	A	-0.0118	0.178	0.762	49739
4	186300882	rs72712015	A	G	-0.0124	0.232	0.790	47104
4	186294284	4:186294284:CT	C	T	0.0067	0.288	0.835	40506
4	186294825	rs3797028	C	T	0.0067	0.288	0.835	41047
4	186295175	rs17884285	G	A	0.0067	0.288	0.835	41397
4	186297012	rs73029523	G	A	0.0067	0.288	0.835	43234
4	186298439	rs111293486	A	G	0.0067	0.288	0.835	44661
4	186299996	rs73029530	G	A	0.0065	0.297	0.855	46218
4	186272721	rs61731371	G	A	0.0064	0.302	0.861	18943
4	186286750	rs28685090	C	T	0.0060	0.305	0.864	32972
4	186290041	rs76146932	G	A	-0.0085	0.315	0.872	36263
4	186293107	rs75148901	G	A	-0.0085	0.315	0.872	39329
4	186293184	rs77535313	G	A	-0.0085	0.315	0.872	39406
4	186301002	rs58462956	T	C	0.0054	0.385	0.929	47224
4	186301815	rs59759585	G	A	0.0054	0.385	0.929	48037
4	186213339	rs62345568	A	G	0.0059	0.387	0.930	-40439
4	186289236	rs113841837	G	C	0.0054	0.394	0.933	35458
4	186292826	rs3756276	C	A	0.0054	0.394	0.933	39048
4	186254126	rs3733644	G	A	-0.0032	0.406	0.945	348
4	186241310	rs59218646	A	G	0.0051	0.418	0.946	-12468
4	186239793	rs73027790	A	G	0.0051	0.418	0.946	-13985
4	186239742	rs73027788	T	C	0.0051	0.418	0.946	-14036
4	186234191	rs57775388	A	G	0.0051	0.418	0.946	-19587
4	186302406	rs10018215	T	G	-0.0026	0.461	0.996	48628
4	186233737	rs80142345	T	C	0.0037	0.473	0.996	-20041
4	186300702	rs67132570	C	T	-0.0028	0.485	0.996	46924
4	186253257	rs1288571	C	T	0.0042	0.496	0.996	-521
4	186261720	rs2587159	G	T	0.0042	0.496	0.996	7942
4	186265044	rs876547	C	A	0.0042	0.496	0.996	11266
4	186265269	rs1288556	G	A	0.0042	0.496	0.996	11491
4	186298424	rs28621464	A	G	-0.0020	0.497	0.996	44646
4	186298593	rs10007747	A	C	-0.0020	0.497	0.996	44815
4	186266309	rs1107715	A	G	-0.0059	0.499	0.996	12531
4	186270045	rs183045841	G	C	-0.0059	0.499	0.996	16267
4	186272178	rs79501274	T	A	-0.0059	0.499	0.996	18400
4	186272183	rs73873429	G	A	-0.0059	0.499	0.996	18405
4	186281719	rs115786770	C	T	-0.0059	0.499	0.996	27941
4	186203972	rs11943671	T	C	-0.0059	0.499	0.996	-49806
4	186205345	rs3108273	A	G	-0.0016	0.523	0.996	-48433
4	186258895	rs72709999	G	A	-0.0036	0.528	0.996	5117
4	186252862	rs10006725	A	G	-0.0026	0.530	0.996	-916
4	186252827	rs10006584	T	C	-0.0026	0.530	0.996	-951
4	186273973	rs11722973	T	C	-0.0022	0.534	0.996	20195
4	186266971	rs73027794	G	A	0.0036	0.566	0.996	13193
4	186278735	rs74735070	C	A	0.0036	0.566	0.996	24957
4	186282104	rs188288011	G	T	0.0036	0.566	0.996	28326
4	186282223	rs1976917	C	T	0.0036	0.566	0.996	28445
4	186204090	rs2692596	T	C	-0.0014	0.581	0.996	-49688
4	186235548	rs2587160	A	G	-0.0023	0.584	0.996	-18230
4	186222890	rs1288559	G	A	-0.0023	0.584	0.996	-30888

4	186249874	rs11728119	A	T	-0.0021	0.608	0.996	-3904
4	186249699	rs11723715	T	C	-0.0021	0.608	0.996	-4079
4	186249485	rs11723704	C	G	-0.0021	0.608	0.996	-4293
4	186287880	rs28428553	C	T	-0.0014	0.612	0.996	34102
4	186288846	rs7664573	G	T	-0.0014	0.612	0.996	35068
4	186254671	rs6838019	G	A	-0.0025	0.614	0.996	893
4	186265634	rs60735992	T	C	-0.0025	0.624	0.996	11856
4	186292169	rs4862522	T	C	-0.0017	0.652	0.996	38391
4	186208715	rs1288534	T	C	-0.0011	0.657	0.996	-45063
4	186274130	rs11727342	G	T	0.0015	0.668	0.996	20352
4	186282359	rs28542795	A	G	-0.0013	0.669	0.996	28581
4	186219938	rs2587162	G	T	-0.0010	0.680	0.996	-33840
4	186218901	rs13109431	G	A	-0.0010	0.680	0.996	-34877
4	186234980	rs28444554	G	A	-0.0021	0.683	0.996	-18798
4	186242051	rs72709996	G	A	0.0024	0.694	0.996	-11727
4	186268576	rs10000918	G	A	-0.0011	0.699	0.996	14798
4	186211966	rs1288541	A	G	-0.0009	0.705	0.996	-41812
4	186276728	rs6820021	C	G	-0.0010	0.726	0.996	22950
4	186271884	rs6811567	C	T	-0.0009	0.731	0.996	18106
4	186275277	rs11941894	G	A	-0.0009	0.731	0.996	21499
4	186281879	rs1963923	G	A	-0.0009	0.731	0.996	28101
4	186232142	rs3806823	T	C	-0.0008	0.734	0.996	-21636
4	186242233	rs10000719	C	T	-0.0012	0.735	0.996	-11545
4	186207367	rs12642709	A	T	-0.0008	0.744	0.996	-46411
4	186207071	rs12642623	C	T	-0.0008	0.744	0.996	-46707
4	186205866	rs12641719	C	T	-0.0008	0.744	0.996	-47912
4	186205791	rs12641710	C	T	-0.0008	0.744	0.996	-47987
4	186204544	rs12640745	C	T	-0.0008	0.744	0.996	-49234
4	186294198	rs3797026	T	C	-0.0010	0.755	0.996	40420
4	186239013	4:186239013:AG	A	G	-0.0012	0.767	0.996	-14765
4	186214271	rs10049586	A	G	-0.0015	0.770	0.996	-39507
4	186272773	rs3822302	C	T	-0.0007	0.786	0.996	18995
4	186229741	rs1288568	C	G	-0.0006	0.786	0.996	-24037
4	186270054	rs11734889	C	G	0.0010	0.789	0.996	16276
4	186269697	rs72712006	T	C	-0.0010	0.790	0.996	15919
4	186267200	rs28557103	A	G	-0.0008	0.791	0.996	13422
4	186298689	rs7670667	G	C	0.0007	0.797	0.996	44911
4	186294998	rs3797029	A	G	-0.0007	0.798	0.996	41220
4	186299519	4:186299519:CA	C	A	-0.0007	0.803	0.996	45741
4	186274541	rs11724024	A	G	-0.0009	0.807	0.996	20763
4	186274922	rs11724164	T	G	-0.0009	0.807	0.996	21144
4	186299758	rs1048329	A	G	-0.0007	0.809	0.996	45980
4	186270641	rs11727513	G	A	-0.0007	0.809	0.996	16863
4	186280804	rs1812114	C	T	-0.0006	0.823	0.996	27026
4	186301125	rs6842078	G	A	-0.0007	0.834	0.996	47347
4	186301988	rs6849261	T	G	-0.0008	0.840	0.996	48210
4	186302038	rs6848938	T	C	-0.0008	0.840	0.996	48260
4	186220378	rs2692593	C	T	-0.0005	0.844	0.996	-33400
4	186224735	rs1288562	T	C	-0.0004	0.853	0.996	-29043
4	186223336	4:186223336:TC	T	C	-0.0004	0.853	0.996	-30442
4	186222638	rs1288557	T	C	-0.0004	0.853	0.996	-31140
4	186269135	rs10023947	T	C	-0.0005	0.854	0.996	15357
4	186271154	rs10030392	A	G	-0.0005	0.854	0.996	17376
4	186283517	rs75662344	A	T	-0.0008	0.855	0.996	29739
4	186252138	rs1288572	C	T	-0.0005	0.857	0.996	-1640
4	186296453	rs10015887	A	C	-0.0005	0.858	0.996	42675
4	186299260	rs2030802	G	A	-0.0005	0.858	0.996	45482

4	186259078	rs75310445	A	G	-0.0007	0.859	0.996	5300
4	186268129	rs11730401	G	A	-0.0006	0.871	0.996	14351
4	186222866	rs1288558	A	G	-0.0004	0.873	0.996	-30912
4	186299985	rs1288548	A	G	-0.0005	0.873	0.996	46207
4	186209982	rs1288536	T	G	-0.0004	0.879	0.996	-43796
4	186279248	rs3816287	G	T	-0.0010	0.881	0.996	25470
4	186274170	rs11727346	C	T	0.0005	0.884	0.996	20392
4	186271550	rs6833759	A	G	-0.0004	0.884	0.996	17772
4	186273488	rs6846997	G	A	-0.0004	0.885	0.996	19710
4	186280522	rs6810778	A	G	-0.0004	0.885	0.996	26744
4	186290246	rs6851813	C	A	-0.0004	0.889	0.996	36468
4	186230276	rs1288569	C	T	0.0003	0.889	0.996	-23502
4	186229268	rs1288567	A	G	0.0003	0.889	0.996	-24510
4	186228540	rs1288565	C	T	0.0003	0.889	0.996	-25238
4	186290508	rs6834425	A	T	-0.0003	0.901	0.996	36730
4	186232081	rs3806822	G	A	-0.0003	0.906	0.996	-21697
4	186283185	rs115309293	T	C	0.0014	0.914	0.996	29407
4	186216136	rs1288547	G	A	-0.0002	0.929	0.996	-37642
4	186209273	4:186209273:GA	G	A	0.0005	0.932	0.996	-44505
4	186284164	rs2120415	T	C	0.0002	0.941	0.996	30386
4	186228877	rs1288566	T	C	-0.0002	0.942	0.996	-24901
4	186271669	rs6810987	C	T	-0.0002	0.954	0.996	17891
4	186272021	rs6834276	C	A	-0.0002	0.954	0.996	18243
4	186272958	rs6841466	A	G	-0.0002	0.954	0.996	19180
4	186281755	rs1031128	C	T	-0.0002	0.954	0.996	27977
4	186294588	rs3822303	G	A	0.0001	0.963	0.996	40810
4	186299656	rs3797032	G	A	0.0001	0.963	0.996	45878
4	186289204	rs28593690	T	C	-0.0002	0.964	0.996	35426
4	186291747	4:186291747:TC	T	C	-0.0002	0.964	0.996	37969
4	186227532	rs753359	G	T	0.0001	0.971	0.996	-26246
4	186292465	rs4861662	G	A	-0.0001	0.975	0.996	38687
4	186287283	rs72712010	C	T	-0.0001	0.976	0.996	33505
4	186287556	rs72712011	C	T	-0.0001	0.976	0.996	33778
4	186287734	rs56326048	C	T	-0.0001	0.976	0.996	33956
4	186267077	rs10034100	T	C	0.0001	0.977	0.996	13299
4	186224535	rs6837989	G	A	0.0001	0.978	0.996	-29243
4	186223289	rs1288560	C	T	0.0001	0.978	0.996	-30489
4	186221375	rs1404997	G	A	0.0001	0.978	0.996	-32403
4	186221369	rs1404996	A	G	0.0001	0.978	0.996	-32409
4	186220035	rs2587163	C	G	0.0000	0.992	0.996	-33743
4	186300030	rs28609143	C	T	0.0001	0.993	0.996	46252

chr = chromosome, position = position of the SNP, SNP = SNP ID, ref = reference allele, alt = alternate allele, beta = regression coefficient, FDR = false discovery rate adjusted p-value, distance = distance of the SNP from the CpG site.

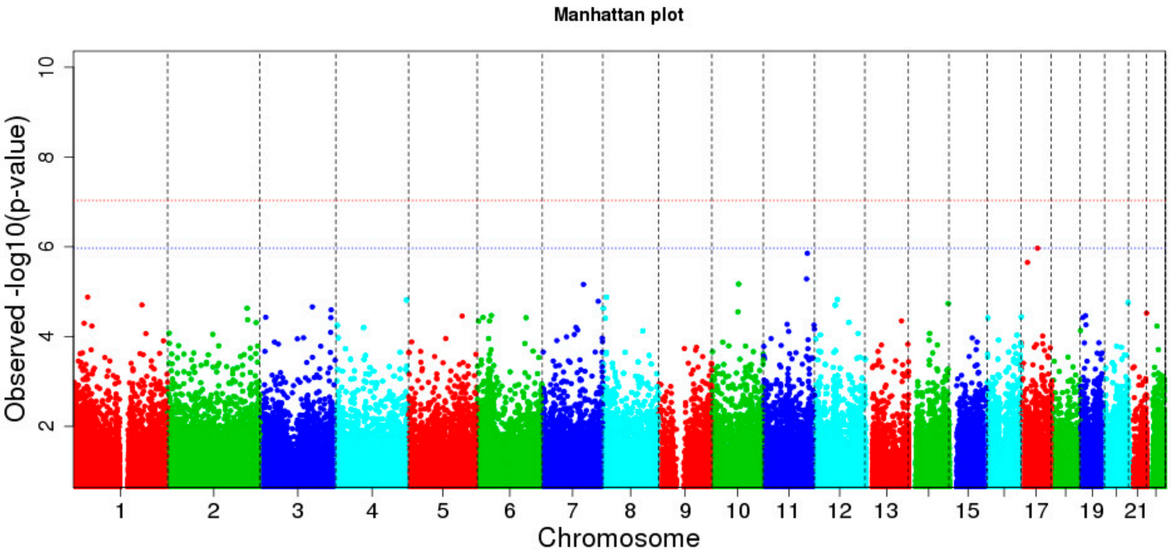

Figure 1. Manhattan plot of the EWAS on breastfeeding duration >3 months and DNAm in the IOWBC at 10 years. The X-axis shows the chromosomes and the Y-axis is the -log10 transformed p-values. The blue and red lines indicate the FDR and Bonferroni thresholds, respectively.

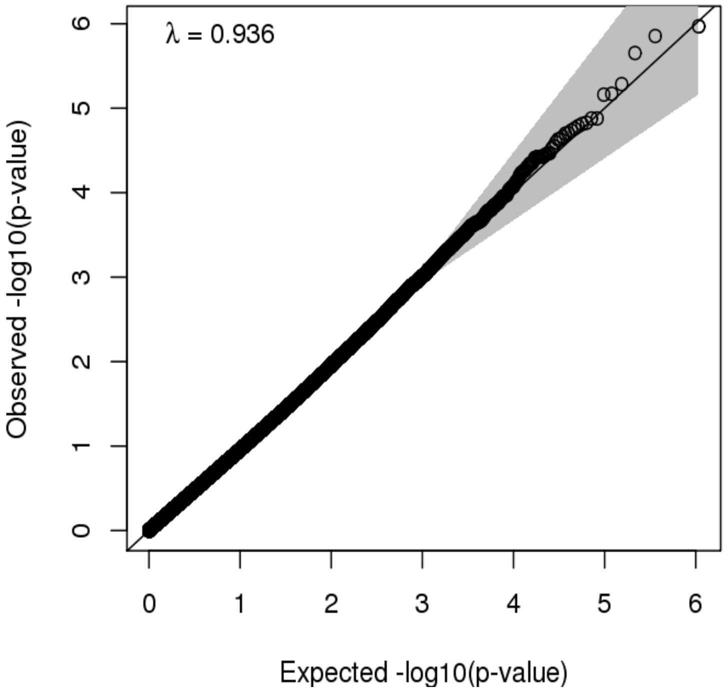

Figure 2. Quantile-Quantile (QQ) plot of the EWAS on breastfeeding duration >3 months and DNAm in the IOWBC at 10 years. Quantile-quantile (Q-Q) plot of observed versus expected (– log 10 (p value)) for the test of association between breastfeeding duration >3 months and DNAm sites.

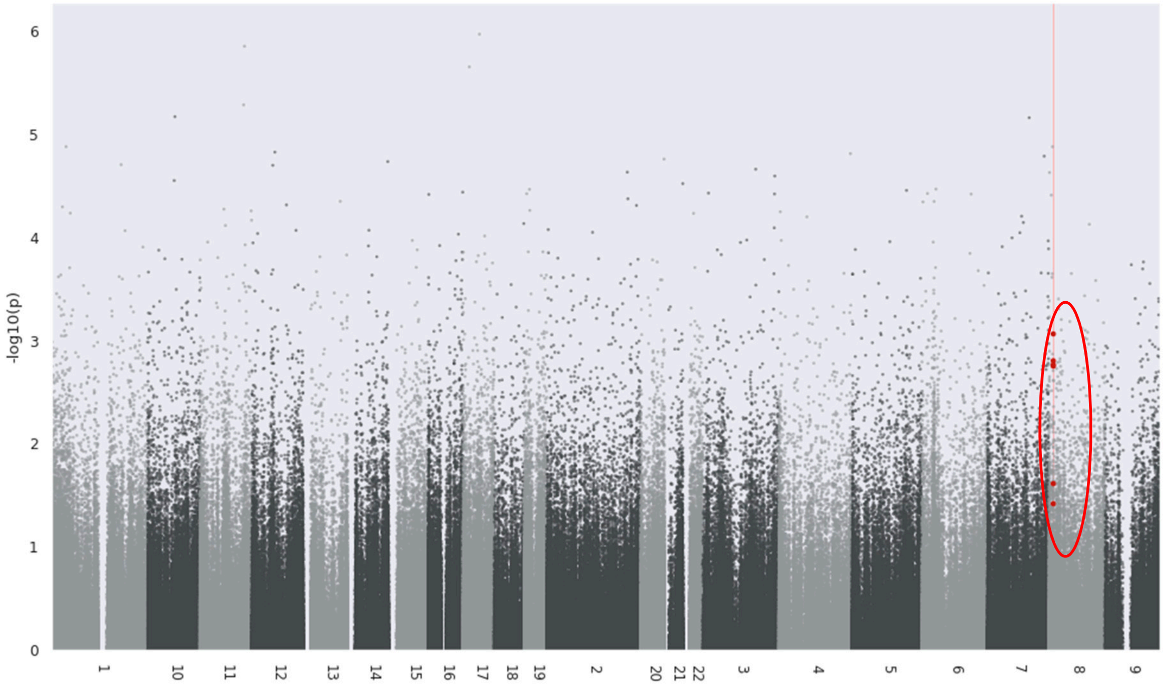

Figure 3. Manhattan plot for the DMR analysis. Manhattan plot of P-values of DMRs identified as significant in the analysis. Regions passing the --region-filter-*p* (<0.05) are highlighted in red.
